# Review of a Case of Paraquat Poisoning in a Tertiary Care Rural-based ICU

**DOI:** 10.5005/jp-journals-10071-23182

**Published:** 2019-06

**Authors:** Deepak S Sharma, Abhishek M Prajapati, Dhruv M Shah

**Affiliations:** 1 Department of Critical Care, King's College Hospital, London, United Kingdom; 2,3 Department of Critical Care, Shree Krishna Hospital, Karamsad, Anand, Gujarat, India

**Keywords:** ARDS, Charcoal hemoperfusion, Paraquat poisoning

## Abstract

Paraquat (1,1′-dimethyl-4, 4′-dipyridylium) is a broad-spectrum liquid herbicide associated with both accidental and intentional ingestion leading to severe and often fatal toxicity.^1^ Paraquat is actively taken up against a concentration gradient into lung tissue leading to pneumonitis and lung fibrosis. Paraquat also causes renal and liver injury.^2^ There are few case publications of paraquat poisoning and only few of them have reported that renal failure has ensued before acute respiratory distress syndrome (ARDS). Our patient presented with above lethal dose intake of paraquat containing substance and we did gastric lavage followed by charcoal hemoperfusion and hemodialysis but patient could not be saved despite optimum efforts suggesting the high fatality of this kind of poisoning.

**How to cite this article:**

Sharma DS, Prajapati AM, Shah DM. Review of a Case of Paraquat Poisoning in a Tertiary Care Rural-based ICU. Indian J Crit Care Med 2019;23(6):284–286.

**Key Messages:**

Acute renal injury with hypoperfusion state due to toxicity at cellular level, redox cycling and intracellular reactive oxidative stress generation may also cause death in early stages in paraquat poisoning despite optimal management.

## INTRODUCTION

This case report is of a young male presented to “Shree Krishna Hospital, Karamsad, Anand” with alleged history of intentional consumption of paraquat containing substance (gramoxone). Despite adequate measures, we lost the patient early but not due to acute respiratory distress syndrome (ARDS), which is the common cause of death in patients with paraquat poisoning. We have searched two of the biggest databases Medline and PubMed and found more than 800 articles, case reports and few case series related to paraquat poisoning. But only less than ten of them mentioned that patient suffered early acute kidney injury before ARDS.

## CASE HISTORY

**Fig. 1:**
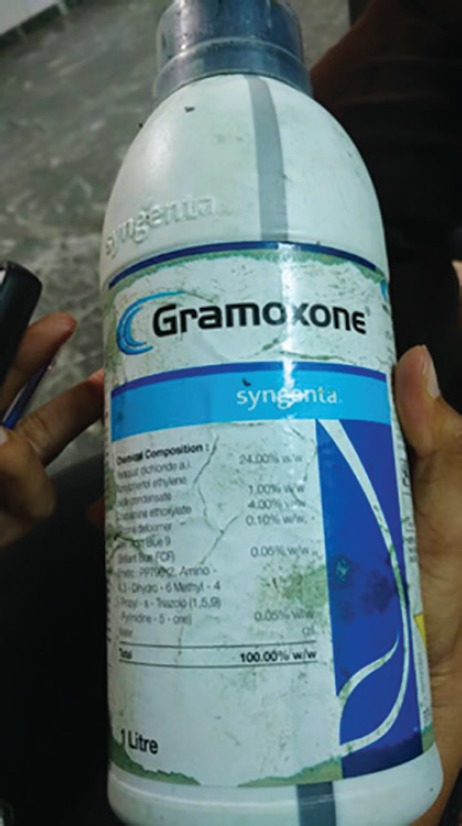
Bottle of paraquat containing liquid gramoxone

**Fig. 2:**
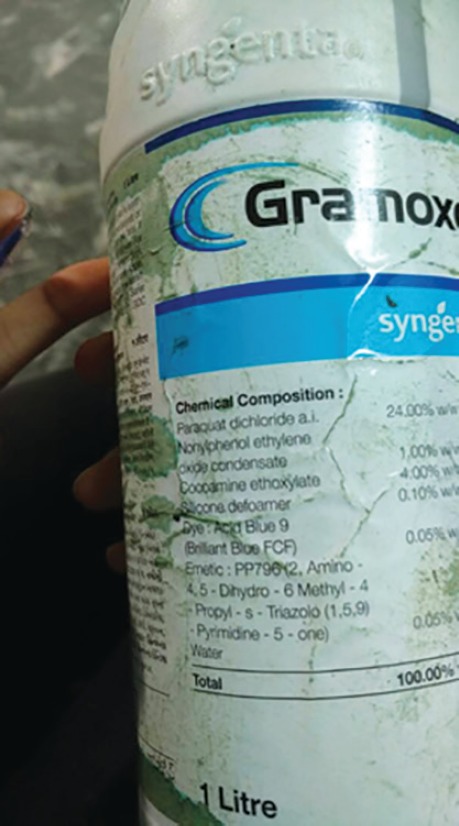
Contents of paraquat containing liquid gramoxane

A 26-year-old male with no known comorbidities presented to trauma and emergency care with alleged history of intentional consumption of around 35 mL of paraquat containing substance (gramoxone) (Figs 1 and 2) at around 8:15 am at his home, which was followed by three episodes of vomiting. There was no history of convulsions, ear nose throat (ENT) bleed or loss of consciousness.

Patient was bought to the emergency room at around 9:30 am and his vital parameters were: heart rate of 100 beats per minute with respiratory rate of 20 breaths per minute, maintaining oxygen saturation of 95% in room air, and blood pressure of 140/80 mm Hg.

### On Systemic Examination

- Respiratory system was clear on auscultation bilaterally and no adventitious sounds were heard,
- Cardiovascular system: S1S2 normal, no rub/gallop/murmur
- Central nervous system: GCS 15/15, pupils bilaterally equally reacting to light, no focal neural deficit
- Abdominal examination: Soft and nontender

### Investigations Trend

Bedside ultrasound abdomen and pelvis was grossly within normal limits. Blood results are shown in Table 1.

As soon as the patient was presented to the emergency room, gastric lavage with KMnO_4_ was given. Patient was then transferred to the intensive care unit where urgent charcoal hemoperfusion and hemodialysis were initiated. Patient remained hemodynamically and neurologically stable during the dialysis. He was also treated with N-acetyl cysteine as antioxidant and other supportive medications. Investigations revealed metabolic acidosis with hyperlactatemia. On the next day, patient progressively developed hypotension along with oliguria. He was intubated in view of deteriorating hemodynamics and was kept on mechanical ventilatory support with vasopressor support. Another session of hemodialysis was done. Patient had persistent hypotension despite high dose vasopressor support and he suffered cardiac arrest at around 12:30 am on the 3rd day of admission, cardiopulmonary resuscitation (CPR) was done as per advanced cardiac life support (ACLS) protocol, but could not be revived and declared dead.

## DISCUSSION

Paraquat (1, 1′-dimethyl-4, 4′-dipyridylium) is a broad-spectrum liquid herbicide associated with both accidental and intentional ingestion leading to severe and often fatal toxicity.^1^ Paraquat generates reactive oxygen species which cause cellular damage via lipid peroxidation, activation of NF-κB, mitochondrial damage, and apoptosis in many organs. Kinetics of distribution into these target tissues can be described by a two-compartment model. Paraquat is actively taken up against a concentration gradient into lung tissue leading to pneumonitis and lung fibrosis. Paraquat also causes renal and liver injury.^2^

The methods to counter poisoning include both hemodialysis and hemoperfusion, anti-inflammatory agents and antioxidants. There have been measures taken to reduce toxicity of paraquat by adding compounds to the herbicide while some countries have banned the herbicide altogether. Diagnosis might be difficult due to lack of proper history and unavailability of diagnostic tests.

Literature review revealed few case reports from India, compared to paraquat poisoning being widely reported from elsewhere. Poisoning with paraquat leads to both local and systemic effects. In an Indian series of 17 patients, the most common symptoms were vomiting (100%), followed by altered sensorium (59%), oral ulceration or dysphagia (53%), dyspnea (41%), or loose stools (24%).^3^ Systemic effects of paraquat are renal and hepatic failure, pulmonary oedema and fibrosis, cardiac failure, shock, convulsions, and multiorgan failure. Involvement of lung in the form of diffuse alveolitis, and subsequent pulmonary fibrosis is the hallmark of paraquat poisoning. Acute respiratory distress syndrome because of paraquat usually appears 24–48 hour after ingestion.^4^

**Table 1:** Blood results

	24/4/17		25/4/17
ABG		5 pm	
pH/ lactate (mmol/L)	7.34/7.9		7.42/2.1
pCO_2_/pO_2_ (mm Hg)	36/101		29/110
HCO_3_ (mmol/L)	19.4		18.8
Base excess (mmol/L)	-5.7		-4.6
S. Na^+^ (mmol/L)	145	140	145
S. K^+^ (mmol/L)	4.5	4.6	2.8
S. cholinesterase (U/L)	16425		
CRP (mg/L)	6.7		
S. creatinine (mg/dL)	1.14	3.40	5.06
TC (x 1000 /µL)	10		8
Hb (g/dL)	13		10.6
PC (/UL)	372,000		100,000
INR/PT	1.01/11.7		1.93/22.80
LFT			
Bilirubin/D/I (mg/dL)	0.72/0.19/0.53		2.12/1.3/0.82
SGPT/SGOT (U/L)	34/38		50/89
S. alk-P (U/L)	80		37
S. amylase			231
Lipase			2508

Poisoning by herbicide paraquat is a common cause of self-poisoning in vast parts of south-east Asia including India.^5^ The very high case fatality due to inherent toxicity and lack of effective treatment is somewhere between 50% and 90%.^6^ Gramoxone contains 24% paraquat dichloride, 1% cocoamine ethoxycar, 0.1% silicone defoamer, 0.05% acid blue dye, PP796, and pyrimidine 5.^7^ Paraquat has been shown to cause significant damage to organs, including the lung, liver, myocardium and kidneys with the highest concentration of paraquat found in the lungs.^8^

A paraquat dose of 30 mg/kg may be fatal, which is equivalent to 8–10 mL of the 20% solution sold commercially.^9^ Our patient was given IV fluid resuscitation of 2.5l bolus followed by 100 mL/hour with normal saline. As patient was not hypoxic initially, so he was not started on oxygen therapy. Initial gastrointestinal decontamination was done with activated charcoal 30 gm in water via a nasogastric tube which is according to published treatment protocols.^2^

Our patient had normal kidney function on the day of admission and underwent hemoperfusion and hemodialysis. In spite of this, the patient developed acute kidney injury on 2nd day of admission. The patient did not improve and developed hypotension on the second day on high dose vasopressors but had cardiac arrest on the third day and expired in spite of CPCR. Patient X-ray was normal throughout the period of hospital admission and patient did not develop hypoxia. These suggest that the patient did not develop acute respiratory distress syndrome, which is the most common cause of death in patients with paraquat poisoning. In our patient, acute kidney injury was the cause of death. Also, in our patient, kidney was involved before the lung, normally either the lung and kidney are involved together or the lung is involved before the kidney.^10^

Another important observation was that in spite of doing hemofiltration, hemodialysis within 4 hours as recommended, patient developed acute kidney injury which led to his death.

## CONCLUSION

In conclusion, our case report emphasizes that it is not only ARDS that kills in paraquat poisoning, renal failure with hypoperfusion state may also cause death in early stages in paraquat poisoning.
